# Attenuation of myogenic orofacial nociception and mechanical hypersensitivity by viral mediated enkephalin overproduction in male and female rats

**DOI:** 10.1186/s12883-015-0285-5

**Published:** 2015-03-15

**Authors:** Phillip R Kramer, Mikhail Umorin, Larry L Bellinger

**Affiliations:** Department of Biomedical Sciences, Texas A&M University Baylor College of Dentistry, 3302 Gaston Avenue, Dallas, TX 75246 USA

**Keywords:** Orofacial, Pain, Temporomandibular joint disorders, Enkephalin, Virus, Masseter muscle

## Abstract

**Background:**

Clinical studies have tested the use of an engineered herpes virus to treat pain. We hypothesized that subcutaneous injections of an engineered herpes virus that expresses enkephalin would attenuate orofacial nociception and hypersensitivity in male and female rats by a central mechanism.

**Methods:**

Herpes virus was injected subcutaneously around the mouth of male and female rats seventy-two hours before ligatures were placed on the masseter tendon, control treatment groups received either no virus or no ligature. Enkephalin expression was measured and von Frey filament testing and meal duration were utilized to measure mechanical hypersensitivity and the nociceptive response, respectively. Naloxone or naloxone methiodide was administered to rats injected with the enkephalin expressing virus to test if enkephalin was acting peripherally or centrally.

**Results:**

Ligature significantly lengthened meal duration and reduced the threshold to von Frey filaments for 18 days. Infection with the enkephalin transgene significantly decreased this response for at least 11 days but only in male rats. Virus injection significantly increased expression of enkephalin in the mental nerve that innervates the mouth region, the trigeminal ganglia and the trigeminal nucleus caudalis but no increase was observed in the masseter nerve after virus injection. Naloxone but not naloxone methiodide reversed the response to the enkephaline expressing virus.

**Conclusions:**

The data suggests that sex should be a considered when using this virus and that viral transfection of the mental nerve with an enkephalin transgene can reduce nociception and hypersensitivity through a central mechanism.

**Electronic supplementary material:**

The online version of this article (doi:10.1186/s12883-015-0285-5) contains supplementary material, which is available to authorized users.

## Background

Chronic orofacial pain such as trigeminal neuralgia, atypical face pain and temporomandibular joint (TMJ) pain are often refractory to current treatment. Control of trigeminal neuralgia pain with drugs is effective in some patients [1] but the effectiveness becomes diminished over time or the patient has side effects to the drugs [2]. Surgery is another option but surgery is not effective in 25-30% of trigeminal neuralgia patients [3,4]. Treatment of TMJ pain by surgical intervention has limitations and additional treatment options are necessary [5,6].

A current pain treatment modality using herpes simplex type I virus to target neuronal expression of enkephalin is currently in clinical trials [7]. Examples of this method’s effectiveness have been demonstrated in an infraorbital nerve ligature model and a facial inflammatory model [8,9]. Current research is testing viral expression vectors to enhance enkephalin concentrations and treat pain [10,11].

The basis for treatment with herpes simplex type I virus is that it attaches to the cell membrane of nerve terminals, internalizes and then retrogradely transports along axons of afferent neurons to the cell body where the viral genome is expressed subsequently affecting cell function [12]. Using this process genes can be engineered into the viral genome for transient expression in host neurons [13]. Wilson et al., 1999 showed that a subcutaneous injection of an engineered virus results in expression of a transgene in sensory neurons innervating the injection site. After infection expression of the transgene occurs within 15 hours [14]. The transfection of sensory neurons with engineered herpes virus has been demonstrated for the dorsal root ganglia and trigeminal ganglia [8,15]. Herpes virus transfection can be used to cause the overexpression of enkephalin and GABA in the dorsal root ganglia to reduce persistent nociception [15-17].

It is unclear if this viral treatment affects men and women differently. This is important because females report a higher amount of orofacial pain than men [18]. For example, women report trigeminal neuralgia and atypical facial pain two to three times more often than men [19] and seek treatment for temporomandibular joint disorders (TMD) more often than men; such that they comprise over three-fourths of the clinical cases. Recently it has been shown that polymorphisms in the estrogen receptor will increase the risk of women developing TMD, such that, woman have a significantly higher risk of moderate or severe pain when polymorphisms are present in this receptor [20,21]; supporting a biological basis for the effect of sex on TMD [22]. Evidence suggests the orofacial pain response in men and women is different because of changes in opioid signaling [23-25]. The opioid enkephalin is affected by sex steroids in certain regions of the female rat brain [26,27]. To date, no study has utilized a chronic animal model to study sex differences in myogenic nociception while altering proenkephalin expression using a viral vector in male and female rats.

It is unknown if the enkephalin viral vector, such as used in this study, has the same efficacy in attenuating orofacial nociception in males and females nor is it clear the location of enkephalin overexpression. Since enkephalin overexpression can result in a reduction of the nociceptive response, locating the enkephalin overexpressing neurons would give clues to the pathway responsible for attenuation. In the present experiments an engineered herpes virus was injected around the mouth and a ligature was placed around the masseter tendon to induce a persistent myogenic response (i.e., TMJ myogenic pain model) in both male and female rats. Mechanical hypersensitivity and the nociceptive response were measured using a von Frey filament assay [28] and a meal duration assay [29,30]. Opioid antagonists naloxone and naloxone methiodide were administered to test enkephalin action peripherally and centrally because naloxone methiodide is impermeable to blood brain barrier. The expression of enkephalin was measured in the mental nerve (the site of injection), the masseter nerve (the site of ligature) and regions where these nerves project; the trigeminal ganglia and the trigeminal nucleus caudalis [31,32].

## Methods

The studies were divided into four experiments; Experiment #1 determined the effect of ligature on meal duration in both males and female rats. Experiment #2 tested the effect of enkephalin on the orofacial nociceptive response in male and female rats by injecting enkephalin producing virus in the mouth region. Experiment #2 also measured enkephalin expression in the trigeminal ganglia 18 days after ligature, the final time point in which von Frey mechanical sensitivity testing was performed, see Table 1. Experiment #3 measured enkephalin expression in both peripheral and central locations when the nociceptive (meal duration) response was attenuated by virus (4 days post injection). More specifically, male rats were injected with virus, ligatured and the mental, masseter nerves, trigeminal ganglia and trigeminal nucleus caudalis were isolated (Table 1). Experiment #4 tested whether enkephalin was acting in the peripheral or central nervous system to attenuate the behavioral pain response. To perform this test opioid receptor antagonists naloxone or naloxone methiodide were administered by osmotic pumps. Naloxone methiodide does not easily cross the blood brain barrier and tests antagonistic effects specific to the peripheral nervous system.

**Table 1:** **Time line for experiments**

Day	Treatment
	**Experiment #1**
−13	▪ Male Sprague–Dawley rats from Harlan Industries arrive
−8	▪ Rats placed in the feeder chambers
−1	▪ A ligature is placed around the tendon of the masseter muscle, the sham group has the tendon exposed but no ligature is placed
11	▪ Rats are sacrificed
	**Experiment #2**
−16	▪ Male Sprague–Dawley rats from Harlan Industries arrive
−13	▪ Gentled for filament testing
−8	▪ Rats placed in the feeder chambers
−7	▪ von Frey filament testing is performed
−4	▪ Innoculate regions around the mouth with five 3 μl injections of vehicle, SHZ virus (control virus), or SHPE virus (enkephalin expressing virus)
−1	▪ von Frey filament testing is performed before surgery
	▪ A ligature is placed around tendon of the masseter muscle for those rats in the chronic pain group, the sham group has the tendon exposed but no ligature is placed
4	▪ von Frey filament testing is performed
	▪ Rats removed from feeders after the enkephalin transgene effect on meal duration was no longer significant
11	▪ von Frey filament testing is performed
18	▪ von Frey filament testing is performed
	▪ Rats are sacrificed and the trigeminal ganglia is isolated for ELISA
	**Experiment #3**
−13	▪ Male Sprague–Dawley rats from Harlan Industries arrive
−4	▪ Innoculate regions around the mouth with five 3 μl injections of vehicle, SHZ virus, or SHPE virus
−1	▪ A ligature is placed around the tendon of the masseter muscle
1	▪ Rats are sacrificed and tissue is isolated for RIA
	**Experiment #4**
−13	▪ Male Sprague–Dawley rats from Harlan Industries arrive
−10	▪ Gentled for filament testing
−8	▪ Rats placed in the feeder chambers
−4	▪ Osmotic pumps surgically implanted subcutaneously contained either vehicle, naloxone or naloxone methiodide
−1	▪ Gentled for filament testing
	▪ Innoculate regions around the mouth with five 3 μl injections of SHPE virus
3	▪ A ligature is placed around the tendon of the masseter muscle
7	▪ von Frey filament testing is performed

### Animal husbandry

The Texas A&M University Baylor College of Dentistry Institutional Animal Care and Use Committee approved the experimental protocol. Male (300 gram) and female (280 gram) Sprague–Dawley rats from Harlan Industries, Houston, TX were kept on a 14:10 light/dark cycle with lights on at 06:00 hours. They were acclimated to the feeding modules for four days before surgery. The rats were given chow pellets and water ad libitum.

### Osmotic pump surgery and drug administration

For surgery rats were anesthetized with 60% of the normal surgical dose of ketamine (52 mg/kg) and xylazine (5.4 mg/kg). Using sterile technique anesthetized rats were implanted with 14-day Alzet mini-osmotic pump that dispensed 12 μl/day of 0.9% saline (vehicle) or 3 mg per kg per day of naloxone or naloxone methiodide [8].

### Virus construction and transfection

**Figure 1:**
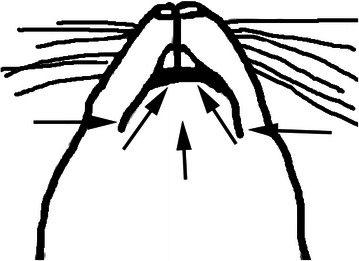
**Cartoon showing at each site, the five locations where vehicle or virus was injected into each rat.**

Replication incompetent herpes simplex I virus was engineered to contain either a LacZ control construct (SHZ) or a construct (SHPE) that expressed human 5 met and 1 leu-enkephalin under the control of the human cytomegalovirus immediate–early promoter inserted in the HSV thymidine kinase locus. The viral vectors were generated as previously described [17] at a concentration of 3.2 × 10^9^ and 4.0 × 10^9^ pfu/ml, respectively. No viral injection was performed in Experiment #1 but subcutaneous injections were completed in Experiment #2, #3 and #4 (see Table 1). Virus expressing SHPE or the SHZ were injected into the rats using a Hamilton syringe after the rats were anesthetized with isoflurane gas 5% / 95% O_2_. Vehicle (i.e., PBS) was injected as a control. Three microliters were injected at each site, into the corners of the mouth, into the lower lip (bilaterally) and into the chin (Figure 1). These regions are innervated by the mental nerve which sends axons directly to the V3 of the trigeminal ganglia [33].

### Ligature placement

Seventy two hours after virus injection (no virus in experiment #1) a ligature was placed around the tendon of the anterior superficial portion of the masseter muscle (TASM); Experiments #1, #2, #3 and #4. TASM results in inflammatory mediator release from the surrounding tissues that enhance the activity of adjacent neurons [28,34]. This model has a myogenic component similar to tenomyositis and muscle pain observed in a human TMD patient [35-37]. Like a human TMD patient where inflammation of the masseter muscle occurs at its attachment to the zygomatic arch (i.e., tenomyositis or tendomyositis) [38,39]. This model has been show to induce a nociceptive response for at least 8 weeks [28], as measured by the rat’s response to press of the skin over the ligatured tendon with von Frey filaments.

In Experiment #1 ligature(s) were given either unilaterally or bilaterally to determine if a nociceptive dose response could be measured. Thus, in a subset of males two 4.0 chromic gut ligatures (Butler Schein, Dublin OH, Cat# 038727), spaced approximately 3.0 mm apart were placed randomly on the masseter tendon on a single side of the head (Table 1). In a different group of male rats a bilateral ligature of the TASM was completed by placing two 4.0 chromic gut ligatures, spaced approximately 3.0 mm apart around the tendon on both sides of the head [28]. Female rats were given bilateral ligatures in Experiment #1. In Experiment #2, #3 and #4 only bilateral ligatures were placed. The incision in the mouth was closed with a single 5.0 polyglycolic acid suture using a 13 mm 3/8 needle (Butler Schein Cat# 493A). Sham operated rats received the same surgery but the tendon was not ligated.

### Meal duration assay

Meal duration can be used as a non-invasive biological marker for TMJ nociception for up to 19 days [29]. Male and female rats with TMJ arthritis eat more slowly, which significantly lengthens the meal duration and pharmacological intervention that reduces pain and inflammation returns the meal duration to normal [40-42]. Patients experiencing TMD pain also have longer chewing cycles and cycle length [43-45]. The lengthening of meal duration during TMD pain [35] is a “guarding behavior”, which can be argued as an operationally defined nociceptive behavior [46].

For the meal duration assay the rats were housed individually in sound-attenuated chambers equipped with photobeam computer-activated pellet feeders (Med Assoc. Inc., East Fairfield, VT) loaded with 45 mg rodent chow pellets (Product No. FO 165, Bioserv, Frenchtown, NJ). When a rat removed a pellet from the feeder trough, a photobeam placed at the bottom of the trough was no longer blocked, signaling the computer to drop another pellet. The computer recorded the date and time and kept a running tally of the total daily food consumption. In these analyses, a meal was defined using a 10-min end of meal criterion (i.e., a meal was bracketed before and after by a 10 minute period of no pellets being taken) and the minimum meal size was set at two pellets [47]. In Experiment #1 (see Table 1) rats were placed in the feeders one week before the ligature surgery and the rats remained in the feeders for 10 days after ligature. In Experiment #2 and #4 the rats were placed in the feeders one week before ligature surgery but remained in the feeder for 4 days after ligature. The daily meal duration was then calculated using Med Assoc. Inc. and proprietary software. The number of animals in each group is shown in Table 2.

**Table 2:** **Treatment Groups and animal numbers**

Masseter surgery	Injection/pump solution	Sex	# of rats
**Experiment #1**			
Sham surgery	No injection	male	12
Unilateral ligature	No injection	male	10
Bilateral ligature	No injection	male	12
Sham surgery	No injection	female	12
Bilateral ligature	No injection	female	12
**Experiment #2**			
Sham surgery	vehicle	male	8
Bilateral ligature	vehicle	male	8
Sham surgery	control virus (SHZ)	male	6
Bilateral ligature	control virus (SHZ)	male	6
Sham surgery	enkephalin virus (SHPE)	male	6
Bilateral ligature	enkephalin virus (SHPE)	male	6
Sham surgery	vehicle	female	8
Bilateral ligature	vehicle	female	8
Sham surgery	control virus (SHZ)	female	6
Bilateral ligature	control virus (SHZ)	female	6
Sham surgery	enkephalin virus (SHPE)	female	6
Bilateral ligature	enkephalin virus (SHPE)	female	6
**Experiment #3**			
Bilateral ligature	control virus (SHZ)	male	8
Bilateral ligature	enkephalin virus (SHPE)	male	8
**Experiment #4**			
Bilateral ligature	enkephalin virus (SHPE)/ vehicle pump	male	6
Bilateral ligature	enkephalin virus (SHPE)/ naloxone pump	male	6
Bilateral ligature	enkephalin virus (SHPE)/ naloxone methiodide pump	male	6

### Filament testing

von Frey filament testing was completed in Experiment #2 and #4. In these tests the animals were gentled by handling a week before filament testing. The filament tests were performed using a series of calibrated von Frey filaments applied to the skin above the masseter tendon (see Table 1 for testing timeline). An active withdrawal of the head from the probing filament was defined as a response. Each von Frey filament was applied five times at intervals of a few sec. The response frequencies (EF50) were calculated as described by Ren’s group [28]. Briefly, the response frequencies [(number of responses/number of stimuli) × 100%] to a range of von Frey filament forces were determined and a stimulus–response frequency curve was plotted. After a non-linear regression analysis, the half maximal response [i.e., EC50 values calculated by Prism 5.0 software (GraphPad, Inc.), here termed EF50] value was derived from the stimulus response curve.

### Tissue isolation, ELISA and RIA assay

No tissue was isolated in Experiment #1. In Experiment #2 the trigeminal ganglia was isolated 18 days after ligature. In Experiment #3 the mental and masseter nerves were isolated along with the trigeminal ganglia and trigeminal nucleus caudalis four days after virus infection (Table 1). Note: the mental nerve is adjacent to the site of injection. The masseter nerve innervates the ligature site and the masseter and mental nerves project to the trigeminal ganglia and then to the caudalis [31,32].

The trigeminal ganglia from each rat just rostral of V1 and 2 mm caudal of V3 was dissected after removal of the brain. To isolate the caudalis nuclei a slice of brainstem was collected from a tissue block that included a 2-mm segment beginning 4–5 mm caudal to the obex. This tissue block included the caudal laminated (Vc) and upper cervical spinal cord (C_1_). The tissue block was turned coronally and the superficial portion of the Vc was harvested. For masseter nerve isolation the temporalis, the masseter and the zygomatic arch were exposed. The temporalis and the masseter were cut immediately above and below the zygomatic arch respectively. The temporalis muscle was reflected and the arch was broken as close to the bodies of the zygomatic and temporal bones as possible and removed. This exposed the superior aspect of the infratemporal fossa. Next, the coronoid process of the mandible was broken off with rongeurs and removed. The masseter nerve was within or immediately anterior to the connective tissue capsule of the TMJ since the masseter nerve projects to V3 of the trigeminal ganglia. A vertical incision in the masseter muscle was made to expose more of the nerve branching within the mass of the muscle. The nerve was traced back toward the foramen ovale along the inferior aspect of the temporal bone. The nerve was extracted by cutting the exposed nerve near foramen ovale proximally and within the tissue of the masseter muscle distally. To isolate the mental nerve the skin to the base of the incisors on the inferior aspect of the head, the connective tissue overlying the incisive alveolar processes was cut to the bone in the anteroposterior direction and reflected anteriorly and laterally. The reflection of the connective tissue exposed the mental foramen on the lateral side of the base of the incisive alveolar process with the mental nerve emerging from the foramen. The nerve was exposed by gently teasing apart the connective tissues at the distal end of the nerve. The nerve was extracted by cutting it at the mental foramen proximally and as close to the connective tissue of the skin distally. Each tissue was stored separately in liquid nitrogen until analysis. Tissue was placed in 300 μl of T-Per tissue protein extraction reagent containing Halt Protease Inhibitor and ground (Thermo Scientific, Rockford, IL, Cat#78510). Human enkephalin quantitation was completed on duplicate 100 μl samples of supernatant in Experiment 2 using an ELISA following the manufacturer’s directions (MyBioSource, San Diego, CA, Cat# MBS269873). Human and rat met-enkephalin quantitation was completed on duplicate 100 μl samples of supernatant in Experiment #3 using an RIA following the manufacturer’s directions (Peninsula Laboratories International Inc., San Carlos, CA, Cat# S-2119). In Experiment #2 and Experiment #3 total protein in the supernatant samples of the trigeminal ganglia and trigeminal nucleus caudalis samples was determined using a BCA protein assay (Thermo Scientific, Waltham, WA, Cat# PI-23221). Isolation of the masseter and mental nerve included a variable amount of connective and muscle tissue, thus to standardize the amount of nervous tissue in each sample we quantitated the amount of 68 kDa light neurofilament (NEFL) in each sample by ELISA following the manufactures directions (Novus Biologicals, Littleton CO, Cat# KA1478). Values were given either as the ng of met-enkephalin per mg of total protein or for the masseter and mental nerve ng of met-enkephalin per ng of NEFL.

### Statistics

Filament data was analyzed using a Mann Whitney test. Meal duration and enkephalin concentration and body weights were analyzed by ANOVA with the dependent variables being meal duration or enkephalin concentration or body weight and the independent variables being surgery, substance injected, sex or days (Prizm 5.0, GraphPad Software Inc, La Jolla, CA). Groups with significant main effects were further analyzed by Duncan’s post-hoc test. In Experiment #3 and Experiment #4 the data was analyzed with a t-test.

## Results

### Experiment #1: Ligature model tested in male and females rats

**Figure 2:**
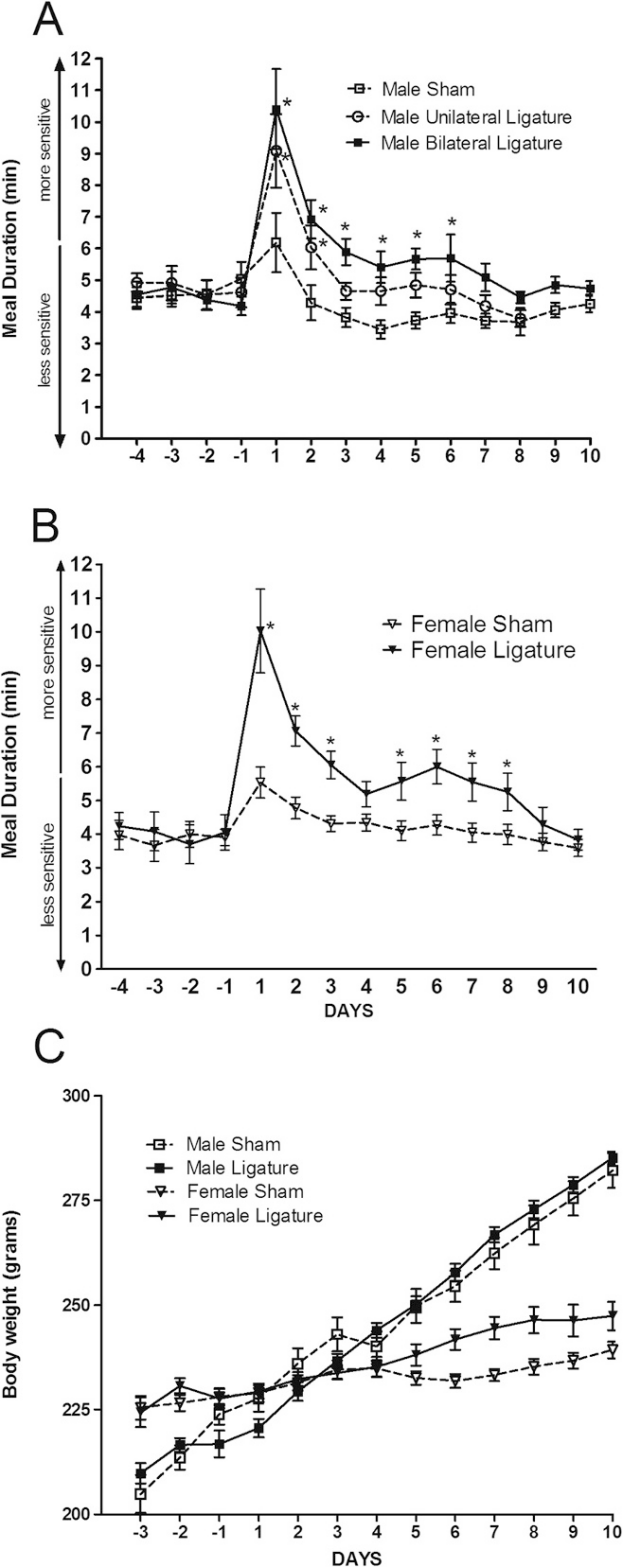
**Experiment #1: Meal duration in male and female rats after masseter tendon ligation.** Sham rats were given anesthetic and the tendon was exposed but no ligature was placed and ligature rats had a tight ligature tied around the tendon of the masseter muscle. In panel **A** the ligature was randomly placed on one side or both sides of a male rat’s masseter tendon to determine a dose response to the surgical treatment and then meal duration was measured. A non-ligatured control group was also included (i.e., sham). In panel **B**, meal duration was measured for female rats with bilateral ligatures. Meal duration values are given for four days before ligature (−4, −3, −2, −1) and for 10 days after ligature (1, 2, 3, 4, etc.). Body weight **(panel C)** was measured daily for the male and female rats. Values are the means ± SEM. Significant differences between the ligature groups and their respective sham groups are indicated by * = p < 0.05. See Table 2 for the number of animals per group.

Ligation resulted in a significant increase in meal duration for both the male F(2, 386) = 21.42, p < 0.01 (Figure 2A, six day increase) and female rats F(1, 352) = 39.65, p < 0.01 (Figure 2B, eight day increase) with a significant interaction between days and surgery for both the males F(22, 386) = 2.17, p < 0.01 (Figure 2A) and females F(13, 352) = 3.1, p < 0.01 (Figure 2B). No significant difference in meal duration was observed between the males and females. To test the dose response of placing a ligature, male rats had either both tendons ligatured or a single tendon ligatured. Rats that had both tendons ligatured had a significantly longer meal duration versus rats that had a ligature placed on only a single side F(1, 231 = 5.43), p < 0.05 with no interaction between days and surgery F(11, 231) = 0.64, p > 0.79 (Figure 2A).

Meal number, meal size, food intake and body weight were not reliable indicators for nociception after TASM ligature (data not shown) consistent with results from rats with TMJ inflammation [29] and from rats with an exposed pulp [29,48].

Body weight values were statistically analyzed and the results show that ligature did not significantly alter bodyweight in either the male or female groups [Figure 2C; F(1,89) = 1.16, p = 0.28]. As expected, the female rats did have a significantly lower body weight gain in comparison to the male rats [Figure 2C; F(1,89) = 32.3, p < 0.001].

### Experiment #2: Enkephalin transgene expression increased and reduced the nociceptive response in male and female rats

**Figure 3:**
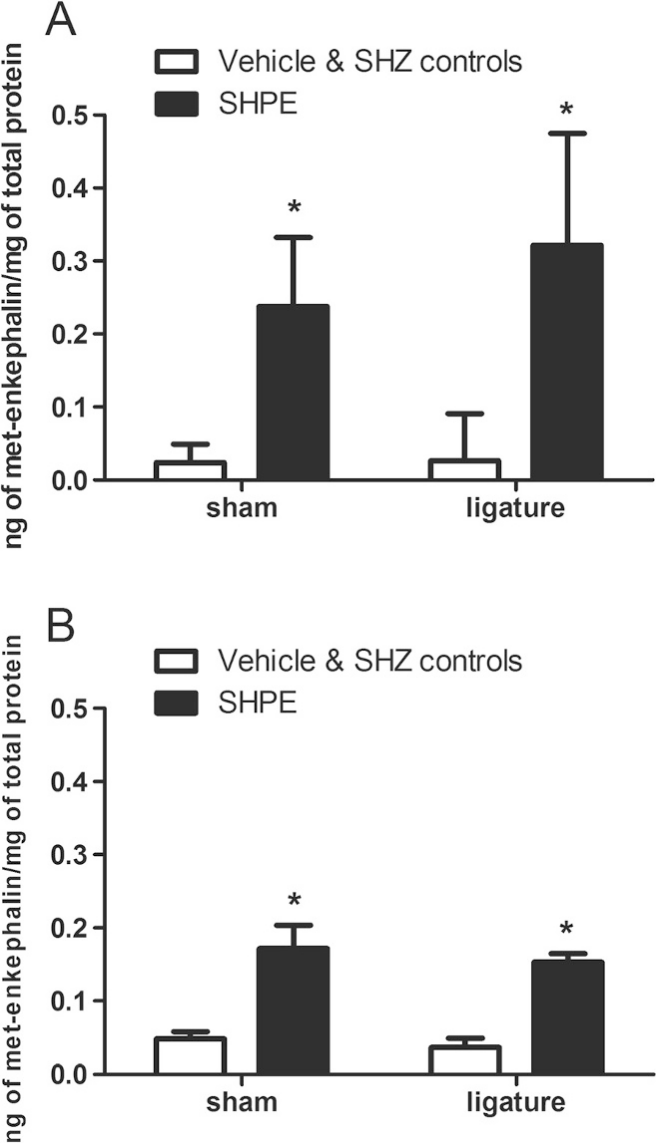
**Experiment #2: Human met-enkephalin in the rat trigeminal ganglia after injection of herpes virus expressing the enkephalin transgene or a control virus.** A single randomly chosen trigeminal ganglia was removed three weeks after injection (see Experiment 2, Table 1) and the human enkephalin protein was quantitated by ELISA. Measurements were made in both male **(panel A)** and female **(panel B)** rats. Groups received either a vehicle injection or an injection of virus that did not contain an enkephalin transgene (control virus, SHZ) or a virus that had an enkephalin transgene (SHPE). The vehicle and SHZ control groups were not significantly different than the zero ng met-enkephalin standard provided in the ELISA kit and the values obtained for these groups were considered background and combined in Figure 3A and 3B. Values are the means ± SEM. An asterisk indicates a significant difference (p < 0.05) between the vehicle & SHZ control groups versus the SHPE virus group. See Table 2 for the number of animals per group.

The amount of human enkephalin in the trigeminal ganglia was significantly higher in the male F(1,19) = 9.0, p < 0.01 (Figure 3A) and female F = (1,26) = 47.7, p < 0.001 (Figure 3B) rats three weeks after injecting the SHPE construct versus rats injected with the vehicle and SHZ control virus. Enkephalin expression in the male trigeminal ganglia was not significantly different than enkephalin expression in the females. No significant interaction was observed between virus injection and ligature surgery for either the males F(1,19) = 0.25, p < 0.61 or females F(1, 26) = 0.03, p < 0.85.

**Figure 4:**
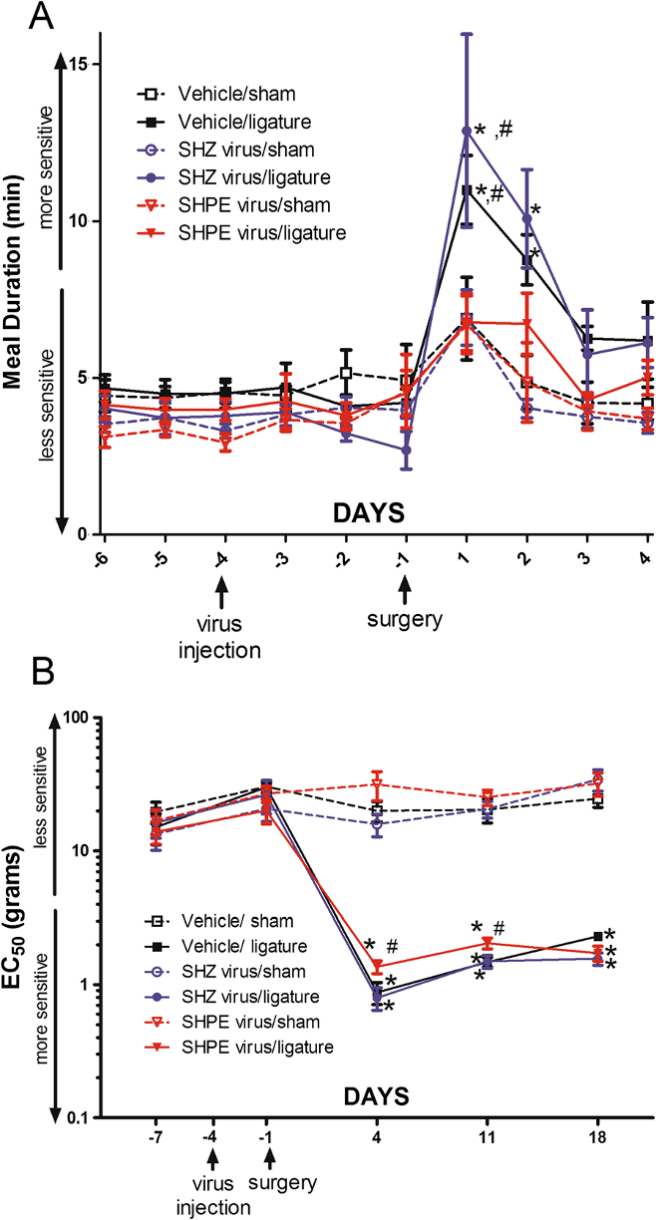
**Experiment #2: Meal duration and von Frey filament testing of male rats after virus injection.** Rats were injected with virus and then 72 hours post-injection a ligature was placed around the tendon of the masseter muscle. Meal duration **(panel A)** was reported for 6 days before ligature surgery (−6, −5, −4, −3, −2, −1) and for 4 days after ligature surgery (1, 2, 3, 3). Filament testing was reported for day 7 before ligature surgery (-7) and 7, 14 and 21 days post-virus injection or 4, 11 and 18 days post-ligature **(panel B)**. Groups received either a vehicle injection or an injection of virus that did not contain an enkephalin transgene (control virus, SHZ) or a virus that had an enkephalin transgene (SHPE) on day −4. In panel **A** the significant differences (p < 0.05) between the vehicle/ligature, SHZ virus/ligature and their respective sham groups are indicated by an asterisk and significant differences (p < 0.05) between the vehicle/ligature and SHZ/ligature versus the SHPE virus/ligature group are indicated by a “#”. In panel **B** significant differences (p < 0.05) between all the ligature groups and their respective sham groups are indicated by an asterisk and significant differences (p < 0.05) between the vehicle/ligature and SHZ/ligature versus the SHPE virus/ligature group is indicated by a “#”. Values are the means ± SEM. See Table 2 for the number of animals per group.

In male rats the meal duration was significantly longer in the vehicle/ligature and SHZ/ligature groups versus their respective sham groups (Figure 4A). The main effect for surgery was F(1,237) = 8.5, p < 0.01 but there was no interaction between surgery and virus injection F(2, 237) = 1.0, p = 0.37. Filament testing revealed a greater injury induced mechanical hypersensitivity in the ligated groups versus the respective sham groups for 18 days post-ligature (Figure 4B). Meal duration was significantly reduced F(2, 204) = 4.8, p < 0.01 in the SHPE virus injected/ligatured rats versus the ligatured rats that received either vehicle or the control SHZ virus (Figure 4A). Upon filament testing a significantly reduced mechanical hypersensitivity was observed in the SHPE virus injected/ligatured rats for 11 days post-injection (Figure 4B).

**Figure 5:**
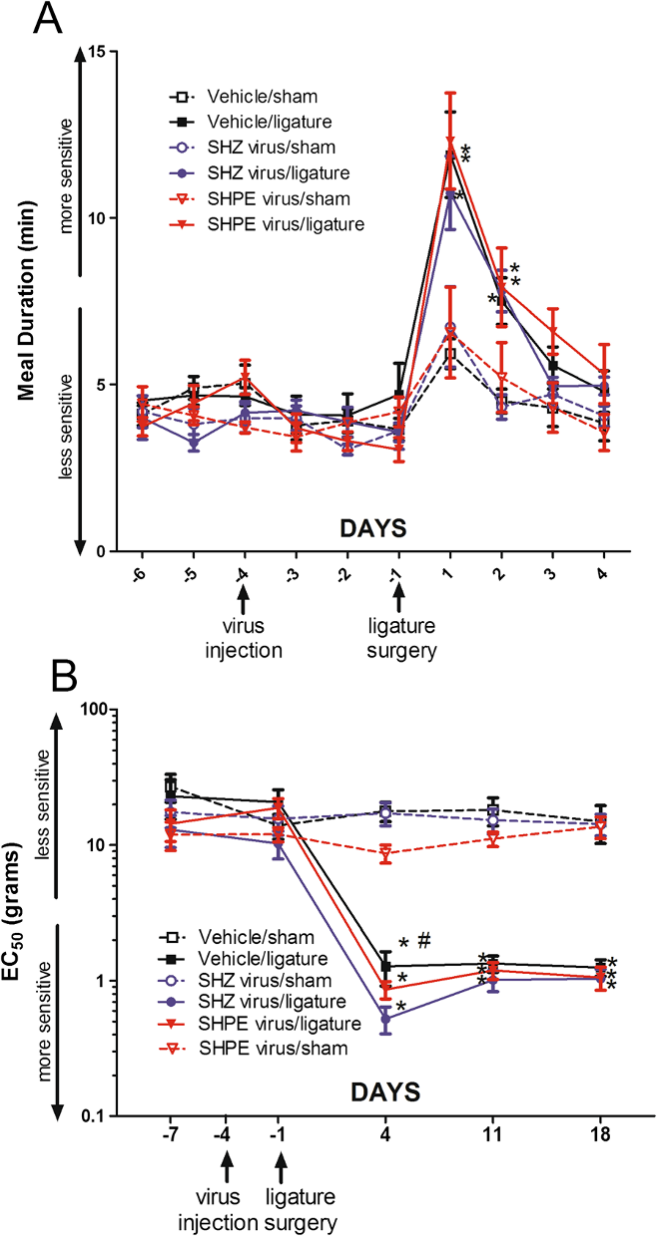
**Experiment #2: Meal duration and von Frey filament testing of female rats after virus injection.** Rats were injected with virus and then 72 hours post-injection a ligature was placed around the tendon of the masseter muscle. Meal duration **(panel A)** was reported for 6 days before ligature surgery (−6, −5, −4, −3, −2, −1) and for 4 days after ligature surgery (1, 2, 3, 3). Filament testing was reported for day 7 before ligature surgery (-7) and 7, 14 and 21 days post-virus injection or 4, 11 and 18 days post-ligature **(panel B)**. Groups received either a vehicle injection or an injection of virus that did not contain an enkephalin transgene (control virus, SHZ) or a virus that had an enkephalin transgene (SHPE) on day −4. Significant differences of (p < 0.05) between the ligature groups and their respective sham groups are indicated by an asterisk and significant differences (p < 0.05) between the vehicle/ligature and SHZ/ligature group is indicated by a “#”. Values are the means ± SEM. See Table 2 for the number of animals per group.

In female rats placing a ligature significantly increased meal duration F(2, 319) = 9.3, p < 0.01 (Figure 5A) but in striking contrast with the male data, treatment with the SHPE virus had no significant effect on the meal duration F(2, 164) = 1.14, p = 0.3. Filament testing indicated there was a significantly greater mechanical hypersensitivity for the 18 day measurement period in female rats after ligature surgery (Figure 5B) which was similar to the males. However, in contrast to the male data, injection with the SHPE virus had no significant effect on attenuating mechanical hypersensitivity (see Additional file 1: Figure S1). Note that injection of virus or surgery did significantly affect the mechanical hypersensitivity (compare the vehicle/ligature and SHZ/ligature groups in Figure 5B).

### Experiment #3: met-enkephalin expression increased in the mental nerve, trigeminal ganglia and trigeminal nucleus caudalis after injection of virus

**Figure 6:**
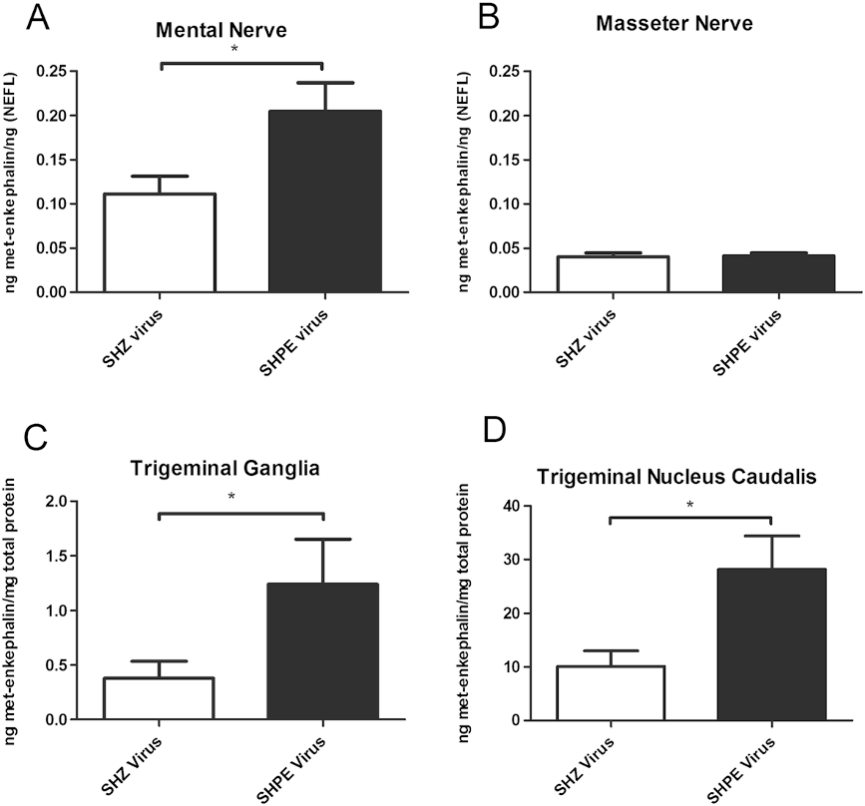
**Experiment #3: Human and rat met-enkephalin in various tissues after injection of herpes virus expressing the enkephalin transgene.** Various neuronal tissues were removed from male rats four days after injection (see Experiment #3, Table 1) and the total amount of enkephalin protein was quantitated by RIA. Measurements were made in the **A)** mental nerve, **B)** masseter nerve, **C)** trigeminal ganglia and **D)** the trigeminal nucleus caudalis. Values are the means ± SEM. An asterisk indicates a significant difference (p < 0.05) between the SHZ control group and the SHPE virus group. See Table 2 for the number of animals per group.

Expression of enkephalin increased significantly in the mental nerve (Figure 6A) but not the masseter nerve (Figure 6B) after injection of SHPE virus. Injection of the enkephalin expressing virus (i.e., SHPE) induced a significant increase in met-enkephalin in both the trigeminal ganglia (Figure 6C) and trigeminal nucleus caudalis (Figure 6D).

### Experiment #4: Opioid antagonists reduced enkephalins attenuation of the allodynic and nociceptive response

**Figure 7:**
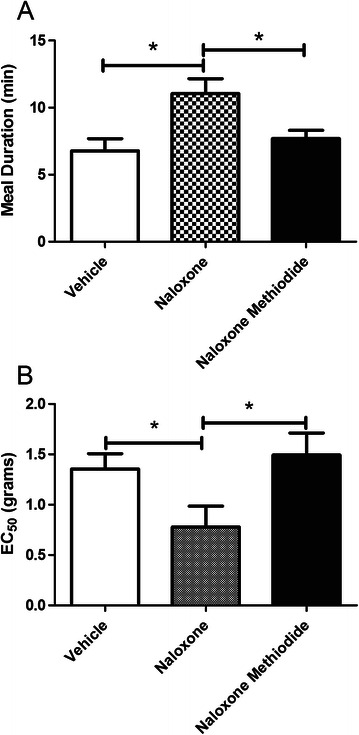
**Experiment #4.** Meal duration and von Frey filament testing of ligatured male rats given opioid antagonist and a viral enkephalin expression vector. Osmotic pumps releasing vehicle, naloxone or naloxone methiodide were surgically implanted subcutaneously in rats. Rats were injected with SPHE virus 72 hours after pump implantation. A ligature was placed around the tendon of the masseter muscle 72 hours after SPHE virus injection. Meal duration **(panel A)** was reported for the day after and von Frey filament testing **(panel B)** was reported on the 4th day after ligature surgery (see Table 1). Significant differences (p < 0.05) between the groups are indicated by an asterisk. Values are the means ± SEM. See Table 2 for the number of animals per group.

Administration of naloxone but not naloxone methiodide lenghthened the meal duration (Figure 7A) and reduced the allodynic threshold (Figure 7B) of ligatures rats injected with enkephalin expressing virus.

## Discussion

The data for Experiment #1 demonstrate that ligation of the TASM can increase nociception and hypersensitivity as measured by two different techniques i.e., meal duration and filament testing thus, confirming the TASM model of Guo et al., [28]. In this study we demonstrate for the first time that the nociceptive response increases as you ligature a greater number of masseter tendons (i.e., bilateral versus unilateral). The data also showed that ligatured females had a significantly longer nociceptive response (i.e., meal duration was lengthened for 8 days) compared to males (6 days), and also extend the ligature model by demonstrating that it is valid for female rats as well as male animals. These data fit well with previous animal studies showing that male rats have a reduced response to muscle stimulation [49] and TMJ inflammation [41,50,51]. Using this model the effect of an enkephalin transgene was different for male and female rats. Enkephalin expression in the trigeminal ganglia of the male rats reduced the nociceptive response after TASM ligature but had no effect in the female rats.

Enkephalin expression in Experiment #2 reduced the response in males but not females when assayed by both meal duration and von Frey testing suggesting enkephalin modulation of the pain response may be different in male and female rats. Measurement of enkephalin expression in the trigeminal ganglia in Experiment #2 indicated that the amount of human enkephalin produced after viral injection was not significantly different between the male and female rats after three weeks. This supports previous studies that indicate herpes virus transport of genetic material is an effective means of introducing gene expression in neurons [52]. The finding that enkephalin expression was similar in the male and female rats is important because enkephalin expression can be modulated by sex steroids in certain regions of the female rat brain [26,27], however we did not observe this sex effect in the trigeminal ganglia. The fact that there was no significant difference could be explained by the strong cytomegalovirus immediate–early promoter driving expression of the enkephalin gene in the viral construct. Enkephalin expression was measured 21 days post-virus injection but future studies would need to address the possibility that enkephalin expression was greater in the males prior to this sampling time (i.e., within the first two weeks after virus injection).

If enkephalin concentration was the same in males and females, consistent with our existing data, then why do females show no significant attenuation of nociception following virus treatment? One possibility is that the delta-opioid system is inherently less active in females as demonstrated by the need for a greater amount delta-opioid receptor agonist to treat orofacial hyperalgesia in female rats [24]. A second possibility is that females have different mu-opioid activity. For example, the trigeminal ganglia contains mu-opioid receptors [53] that when activated reduce nociception in rats with masseter muscle inflammation [54]. Neurons from the trigeminal ganglia project axons that terminate in the trigeminal nucleus caudalis where estrogen can binds its receptor to alter the opioid pathway [55]. This mu-opioid system is attenuated in cycling females when the estrogen levels are at a nadir (i.e., diestrus) [23] but when estrogen levels peak the mu-opioid system has greater activity, equivalent to men [56]. This decrease or increase in the mu-opioid system’s activity can be associated with a respective increase or decrease of the nociceptive response [57,58]. Because enkephalin has a high binding affinity for mu and delta opioid receptors one explanation for our results is that the females had reduced mu or delta-opioid activity in comparison to the males. We suggest this possibility based on studies that show estrogen attenuates antinociception through an opioid pathway [59], possibly mediated by opioid neurons that co-express the estrogen receptor [55,60].

Injections of met-enkephalin producing SHPE virus reduced the nociceptive response but we wanted to determine if the effects were peripheral or central. Orofacial nociceptive responses are affected by an opioid dependent pathway peripherally [25,61]. Experiment #3 was performed for the purpose of determining met-enkephalin expression four days after virus injection; the time when meal duration was significantly attenuated. The experiment was limited to male rats that received ligature to reduce animal numbers. No significant increase in enkephalin was observed in the masseter nerve suggesting the spread of virus was localized to the injection site and peripheral effects were localized to sensory nerves innervating the injected region. Central action of enkephalin was supported by an observed increase in enkephalin in the trigeminal nucleus caudalis. Experiment #4 confirmed this result in that naloxone methiodide, which has difficulty crossing the blood brain barrier, did not reverse the effects of the enkephalin virus whereas naloxone did. A possible mechanism for this effect is by enkephalin binding delta opioid receptors in the caudalis, activating GIRK channels effecting other central sites that cause a attenuation of the masseteric hyperalgesia [62].

Measurement of the enkephalin protein in Experiment #2 was completed by an ELISA assay that had specificity for human enkephalin, note that the SHPE virus expressed the human enkephalin gene. The ELISA may cross react with rat met-enkephalin to a limited degree because we did observe some signal in the SHZ control group. The ELISA had a small amount of background signal as observed in the standard that had zero ng of human met-enkephalin.

Samples in Experiment #2 were near the minimum sensitivity of the ELISA assay thus, in Experiment #3 we choose to utilize a more sensitive RIA assay. The RIA assay detected both human and rat met-enkephalin unlike the ELISA which was reported to be specific for human met-enkephalin. In Experiment #2 the met-enkephalin in the trigeminal ganglia of SHPE injected, ligatured rats increased 0.32 ng ± 0.15 versus controls and in Experiment #3 the met-enkephalin increased 0.8 ng ± 0.4 when comparing the same groups suggesting that, within the error of the assay, the increase in enkephalin after infusion of the SHPE virus was similar as measured by both ELISA and RIA.

A large proportion of TMD in humans involves orofacial muscles [23,63], which appear to be important in producing long lasting and severe orofacial pain. In this regard, the TASM ligature model was an improvement from previous models because it produces pain for days versus models that use formalin or hypertonic saline injections. Thus, the benefits of the TASM model are at least two fold, first the model utilized orofacial muscles which contribute to the pain reported in a majority of TMD patients. Second, the model produced a long lasting nociceptive response versus masseter injection of saline or formalin. TASM ligation results in neuronal activity due, in part, to nociceptive neurons near the tendon (e.g., fascia, muscle and connective tissue) [64] becoming sensitized as the result of biochemical changes in and around the tendon [65,66]. One example of these biochemical changes is an increased MMP-9 [67] resulting in greater sensitivity of the nociceptive neurons in the surrounding tissues [68]. In addition, ligation of the masseter tendon has been shown to increase NMDA receptor expression within the Vc-C_1_ region [28] which corresponded to greater c-fos expression in the trigeminal nucleus and a significantly greater nociceptive response 18 days post treatment.

Body weight was not affected by TASM ligature in either the females or males and the differences were noted solely as the result of sex differences in growth rates. A potential confounder in the feeding model is that ligature of the tendon could stimulate neurons of the Golgi organs to reduce function of the masseter muscle. The fact that the rat’s meal patterns (i.e., food intake, meal frequency and meal size) remained unchanged suggests that that ligation did not inhibit the mechanical feeding processes.

## Conclusions

Sex should be a consideration when utilizing virally mediated enkephalin expression to treat pain. Enkephalin overexpression observed in the trigeminal nucleus caudalis and trigeminal ganglia but not the masseter nerve suggests attenuation of hypersensitivity and the nociceptive response was mediated more centrally.

## Additional file

Additional file 1: Figure S1. Experiment #2: Meal duration for male and female rats one day after ligature surgery. Rats were injected with virus and then 72 hours post-injection a ligature was placed around the tendon of the masseter muscle. Meal duration is reported for the day after surgery. Groups received either a vehicle injection or an injection of virus that did not contain an enkephalin transgene (control virus, SHZ) or a virus that had an enkephalin transgene (SHPE) 72 hours before ligature surgery. A significant difference (p<0.05, t-test) between the male and females was observed in rats that received the SHPE virus (asterisk). See Table 2 for the number of animals per group.

## References

[1] Wiffen P, Collins S, McQuay H, Carroll D, Jadad A, Moore A (2000). Anticonvulsant drugs for acute and chronic pain. Cochrane Database Syst Rev.

[2] Krafft RM (2008). Trigeminal neuralgia. Am Fam Physician.

[3] Sarsam Z, Garcia-Finana M, Nurmikko TJ, Varma TR, Eldridge P (2010). The long-term outcome of microvascular decompression for trigeminal neuralgia. Br J Neurosurg.

[4] Dhople AA, Adams JR, Maggio WW, Naqvi SA, Regine WF, Kwok Y (2009). Long-term outcomes of Gamma Knife radiosurgery for classic trigeminal neuralgia: implications of treatment and critical review of the literature. J Neurosurg.

[5] Manfredini D, Castroflorio T, Perinetti G, Guarda-Nardini L (2012). Dental occlusion, body posture and temporomandibular disorders: where we are now and where we are heading for. J Oral Rehabil.

[6] Marini I, Gatto MR, Bartolucci ML, Bortolotti F, Alessandri Bonetti G, Michelotti A (2013). Effects of experimental occlusal interference on body posture: an optoelectronic stereophotogrammetric analysis. J Oral Rehabil.

[7] Fink DJ, Wechuck J, Mata M, Glorioso JC, Goss J, Krisky D, Wolfe D (2011). Gene therapy for pain: results of a phase I clinical trial. Ann Neurol.

[8] Meunier A, Latremoliere A, Mauborgne A, Bourgoin S, Kayser V, Cesselin F, Hamon M, Pohl M (2005). Attenuation of pain-related behavior in a rat model of trigeminal neuropathic pain by viral-driven enkephalin overproduction in trigeminal ganglion neurons. Mol Ther.

[9] Yeomans DC, Klukinov M (2012). A rodent model of trigeminal neuralgia. Methods Mol Biol.

[10] Wolfe D. Mata M. Targeted drug delivery to the peripheral nervous system using gene therapy. NeurosciLett: Fink DJ; 2012 10.1016/j.neulet.2012.04.047 PMC3458184 22565023

[11] Simonato M, Bennett J, Boulis NM, Castro MG, Fink DJ, Goins WF, Gray SJ, Lowenstein PR, Vandenberghe LH, Wilson TJ (2013). Progress in gene therapy for neurological disorders. Nat Rev Neurol.

[12] Diefenbach RJ, Miranda-Saksena M, Douglas MW, Cunningham AL (2008). Transport and egress of herpes simplex virus in neurons. Rev Med Virol.

[13] Gage PJ, Sauer B, Levine M, Glorioso JC (1992). A cell-free recombination system for site-specific integration of multigenic shuttle plasmids into the herpes simplex virus type 1 genome. J Virol.

[14] Jenkins FJ, Turner SL (1996). Herpes simplex virus: a tool for neuroscientists. Front Biosci.

[15] Chattopadhyay M, Mata M, Fink DJ (2011). Vector-mediated release of GABA attenuates pain-related behaviors and reduces Na(V)1.7 in DRG neurons. Eur J Pain.

[16] Hao S, Mata M, Goins W, Glorioso JC, Fink DJ (2003). Transgene-mediated enkephalin release enhances the effect of morphine and evades tolerance to produce a sustained antiallodynic effect in neuropathic pain. Pain.

[17] Goss JR, Mata M, Goins WF, Wu HH, Glorioso JC, Fink DJ (2001). Antinociceptive effect of a genomic herpes simplex virus-based vector expressing human proenkephalin in rat dorsal root ganglion. Gene Ther.

[18] Fillingim RB, King CD, Ribeiro-Dasilva MC, Rahim-Williams B, Riley JL (2009). Sex, gender, and pain: a review of recent clinical and experimental findings. J Pain.

[19] Koopman JSHA, Dieleman JP, Huygen FJ, de Mos M, Martin CGM, Sturkenboom MCJM (2009). Incidence of facial pain in the general population. Pain.

[20] Kang SC, Lee DG, Choi JH, Kim ST, Kim YK, Ahn HJ (2007). Association between estrogen receptor polymorphism and pain susceptibility in female temporomandibular joint osteoarthritis patients. Int J Oral Maxillofac Surg.

[21] Ribeiro-Dasilva MC, Peres L, Arthuri MT, Hou W, Fillingim RB, Rizzatti Barbosa CM, MC LGdS (2009). Estrogen receptor-alpha polymorphisms and predisposition to TMJ disorder. J Pain.

[22] LeResche L (1997). Epidemiology of temporomandibular disorders: implications for the investigation of etiologic factors. Int J Oral Maxillofac Surg.

[23] Zubieta JK, Smith YR, Bueller JA, Xu Y, Kilbourn MR, Jewett DM, Meyer CR, Koeppe RA, Stohler CS (2002). mu-opioid receptor-mediated antinociceptive responses differ in men and women. J Neurosci.

[24] Saloman JL, Niu KY, Ro JY (2011). Activation of peripheral delta-opioid receptors leads to anti-hyperalgesic responses in the masseter muscle of male and female rats. Neuroscience.

[25] Clemente JT, Parada CA, Veiga MC, Gear RW, Tambeli CH (2004). Sexual dimorphism in the antinociception mediated by kappa opioid receptors in the rat temporomandibular joint. Neurosci Lett.

[26] Lauber AH, Romano GJ, Mobbs CV, Howells RD, Pfaff DW (1990). Estradiol induction of proenkephalin messenger RNA in hypothalamus: dose–response and relation to reproductive behavior in the female rat. Brain Res Mol Brain Res.

[27] Romano GJ, Mobbs CV, Lauber A, Howells RD, Pfaff DW (1990). Differential regulation of proenkephalin gene expression by estrogen in the ventromedial hypothalamus of male and female rats: implications for the molecular basis of a sexually differentiated behavior. Brain Res.

[28] Guo W, Wang H, Zou S, Wei F, Dubner R, Ren K (2010). Long lasting pain hypersensitivity following ligation of the tendon of the masseter muscle in rats: a model of myogenic orofacial pain. Mol Pain.

[29] Kramer PR, Kerins CA, Schneiderman E, Bellinger LL (2010). Measuring persistent temporomandibular joint nociception in rats and two mice strains. Physiol Behav.

[30] Kerins CA, Carlson DS, Hinton RJ, Grogan DM, Marr K, Kramer PR, Spears RD, Bellinger LL (2005). Specificity of meal pattern analysis as an animal model of dermining temporomandibular joint inflammation/pain. Int J Oral Maxillofac Surg.

[31] Marfurt CF (1981). The central projections of trigeminal primary afferent neurons in the cat as determined by the tranganglionic transport of horseradish peroxidase. J Comp Neurol.

[32] Capra NF, Wax TD (1989). Distribution and central projections of primary afferent neurons that innervate the masseter muscle and mandibular periodontium: a double-label study. J Comp Neurol.

[33] Zuniga JR (1999). Trigeminal ganglion cell response to mental nerve transection and repair in the rat. J Oral Maxillofac Surg.

[34] Kramer PR, Bellinger LL (2013). Reduced GABA receptor alpha6 expression in the trigeminal ganglion enhanced myofascial nociceptive response. Neuroscience.

[35] Bailey JO, McCall WD, Ash MM (1977). Electromyographic silent periods and jaw motion parameters: quantitative measures of temporomandibular joint dysfunction. J Dent Res.

[36] Helkimo MI, Bailey JO, Ash MM (1979). Correlations of electromyographic silent period duration and the Helkimo dysfunction index. Acta Odontol Scand.

[37] Franks AS (1965). Masticatory muscle hyperactivity and temporomandibular joint dysfunction. J Prosthet Dent.

[38] DuPont JS, Brown CE (2009). Masseter tenomyositis. Cranio.

[39] Friedman MH, Agus B, Weisberg J (1983). Neglected conditions producing preauricular and referred pain. J Neurol Neurosurg Psychiatry.

[40] Kerins C, Carlson D, McIntosh J, Bellinger L (2004). A role for cyclooxygenase II inhibitors in modulating temporomandibular joint inflammation from a meal pattern analysis perspective. J Oral Maxillofac Surg.

[41] Bellinger LL, Spears R, King CM, Dahm F, Hutchins B, Kerins CA, Kramer PR (2007). Capsaicin sensitive neurons role in the inflamed TMJ acute nociceptive response of female and male rats. Physiol Behav.

[42] Kerins CA, Spears R, Bellinger LL, Hutchins B (2003). The prospective use of COX-2 inhibitors for the treatment of temporomandibular joint inflammatory disorders. Int J Immunopathol Pharmacol.

[43] Hansdottir R, Bakke M (2004). Joint tenderness, jaw opening, chewing velocity, and bite force in patients with temporomandibular joint pain and matched healthy control subjects. J Orofac Pain.

[44] Bakke M, Hansdottir R (2008). Mandibular function in patients with temporomandibular joint pain: a 3-year follow-up. Oral Surg Oral Med Oral Pathol Oral Radiol Endod.

[45] Pereira LJ, Steenks MH, de Wijer A, Speksnijder CM, van der Bilt A (2009). Masticatory function in subacute TMD patients before and after treatment. J Oral Rehabil.

[46] Sternberg WF, Wachterman MW, Fillingim RB (2000). Experimental studies of sex-related factors influencing nociceptive responses: nonhuman animal research. Sex, Gender and Pain.

[47] Castonguay TW, Kaiser LL, Stern JS (1986). Meal pattern analysis: artifacts, assumptions and implications. Brain Res Bull.

[48] Kramer PR, He J, Puri J, Bellinger LL (2012). A Non-invasive Model for Measuring Nociception after Tooth Pulp Exposure. J Dent Res.

[49] Cairns BE, Sim Y, Bereiter DA, Sessle BJ, Hu JW (2002). Influence of sex on reflex jaw muscle activity evoked from the rat temporomandibular joint. Brain Res.

[50] Fischer L, Torres-Chavez KE, Clemente-Napimoga JT, Jorge D, de Arruda Veiga MC, Tambeli CH (2008). The influence of sex and ovarian hormones on temporomandibular joint nociception in rats. J Pain.

[51] Fischer L, Clemente JT, Tambeli CH (2007). The protective role of testosterone in the development of temporomandibular joint pain. J Pain.

[52] Wilson SP, Yeomans DC, Bender MA, Lu Y, Goins WF, Glorioso JC (1999). Antihyperalgesic effects of infection with a preproenkephalin-encoding herpes virus. Proc Natl Acad Sci USA.

[53] Buzas B, Cox BM (1997). Quantitative analysis of mu and delta opioid receptor gene expression in rat brain and peripheral ganglia using competitive polymerase chain reaction. Neuroscience.

[54] Nunez S, Lee JS, Zhang Y, Bai G, Ro JY (2007). Role of peripheral mu-opioid receptors in inflammatory orofacial muscle pain. Neuroscience.

[55] Flores CA, Shughrue P, Petersen SL, Mokha SS (2003). Sex-related differences in the distribution of opioid receptor-like 1 receptor mRNA and colocalization with estrogen receptor mRNA in neurons of the spinal trigeminal nucleus caudalis in the rat. Neuroscience.

[56] Smith YR, Stohler CS, Nichols TE, Bueller JA, Koeppe RA, Zubieta JK (2006). Pronociceptive and antinociceptive effects of estradiol through endogenous opioid neurotransmission in women. J Neurosci.

[57] Bernal SA, Morgan MM, Craft RM (2007). PAG mu opioid receptor activation underlies sex differences in morphine antinociception. Behav Brain Res.

[58] Stoffel EC, Ulibarri CM, Folk JE, Rice KC, Craft RM (2005). Gonadal hormone modulation of mu, kappa, and delta opioid antinociception in male and female rats. J Pain.

[59] Claiborne J, Nag S, Mokha SS (2006). Activation of opioid receptor like-1 receptor in the spinal cord produces sex-specific antinociception in the rat: estrogen attenuates antinociception in the female, whereas testosterone is required for the expression of antinociception in the male. J Neurosci.

[60] Amandusson Å, Hermanson O, Blomqvist A (1996). Colocalization of Oestrogen Receptor lmmunoreactivity and Preproenkephalin mRNA Expression to Neurons in the Superficial Laminae of the Spinal and Medullary Dorsal Horn of Rats. Eur J Neurol.

[61] Arthuri MT, Gameiro GH, Tambeli CH, Arruda Veiga MC (2005). Peripheral effect of a kappa opioid receptor antagonist on nociception evoked by formalin injected in TMJ of pregnant rats. Life Sci.

[62] Chung MK, Cho YS, Bae YC, Lee J, Zhang X, Ro JY (2014). Peripheral G protein-coupled inwardly rectifying potassium channels are involved in delta-opioid receptor-mediated anti-hyperalgesia in rat masseter muscle. Eur J Pain.

[63] Stohler CS (1999). Muscle-related temporomandibular disorders. J Orofac Pain.

[64] Ackermann P, Bring D-I, Renström P, Maffulli N, Renström P, Leadbetter W (2005). Tendon Innervation and Neuronal Response After Injury. Tendon Injuries.

[65] Alfredson H, Forsgren S, Thorsen K, Lorentzon R (2001). In vivo microdialysis and immunohistochemical analyses of tendon tissue demonstrated high amounts of free glutamate and glutamate NMDAR1 receptors, but no signs of inflammation, in Jumper’s knee. J Orthop Res.

[66] Schubert TE, Weidler C, Lerch K, Hofstadter F, Straub RH (2005). Achilles tendinosis is associated with sprouting of substance P positive nerve fibres. Ann Rheum Dis.

[67] Jones GC, Corps AN, Pennington CJ, Clark IM, Edwards DR, Bradley MM, Hazleman BL, Riley GP (2006). Expression profiling of metalloproteinases and tissue inhibitors of metalloproteinases in normal and degenerate human achilles tendon. Arthritis Rheum.

[68] Kawasaki Y, Xu ZZ, Wang X, Park JY, Zhuang ZY, Tan PH, Gao YJ, Roy K, Corfas G, Lo EH (2008). Distinct roles of matrix metalloproteases in the early- and late-phase development of neuropathic pain. Nat Med.

