# Supplementary material for: Attenuation of myogenic orofacial nociception and mechanical hypersensitivity by viral mediated enkephalin overproduction in male and female rats
Source: BMC Neurol. 2015 Mar 15;15:34. doi: 10.1186/s12883-015-0285-5 (PMC4369359; doi:10.1186/s12883-015-0285-5)
Supplement: Additional file 1: Figure S1. — Experiment #2: Meal duration for male and female rats one day after ligature surgery. Rats were injected with virus and then 72 hours post-injection a ligature was placed around the tendon of the masseter muscle. Meal duration is reported for the day after surgery. Groups received either a vehicle injection or an injection of virus that did not contain an enkephalin transgene (control virus, SHZ) or a virus that had an enkephalin transgene (SHPE) 72 hours before ligature surgery. A significant difference (p<0.05, t-test) between the male and females was observed in rats that received the SHPE virus (asterisk). See Table 2 for the number of animals per group. [file 12883_2015_285_MOESM1_ESM.docx]

Additional file1: Figure S1


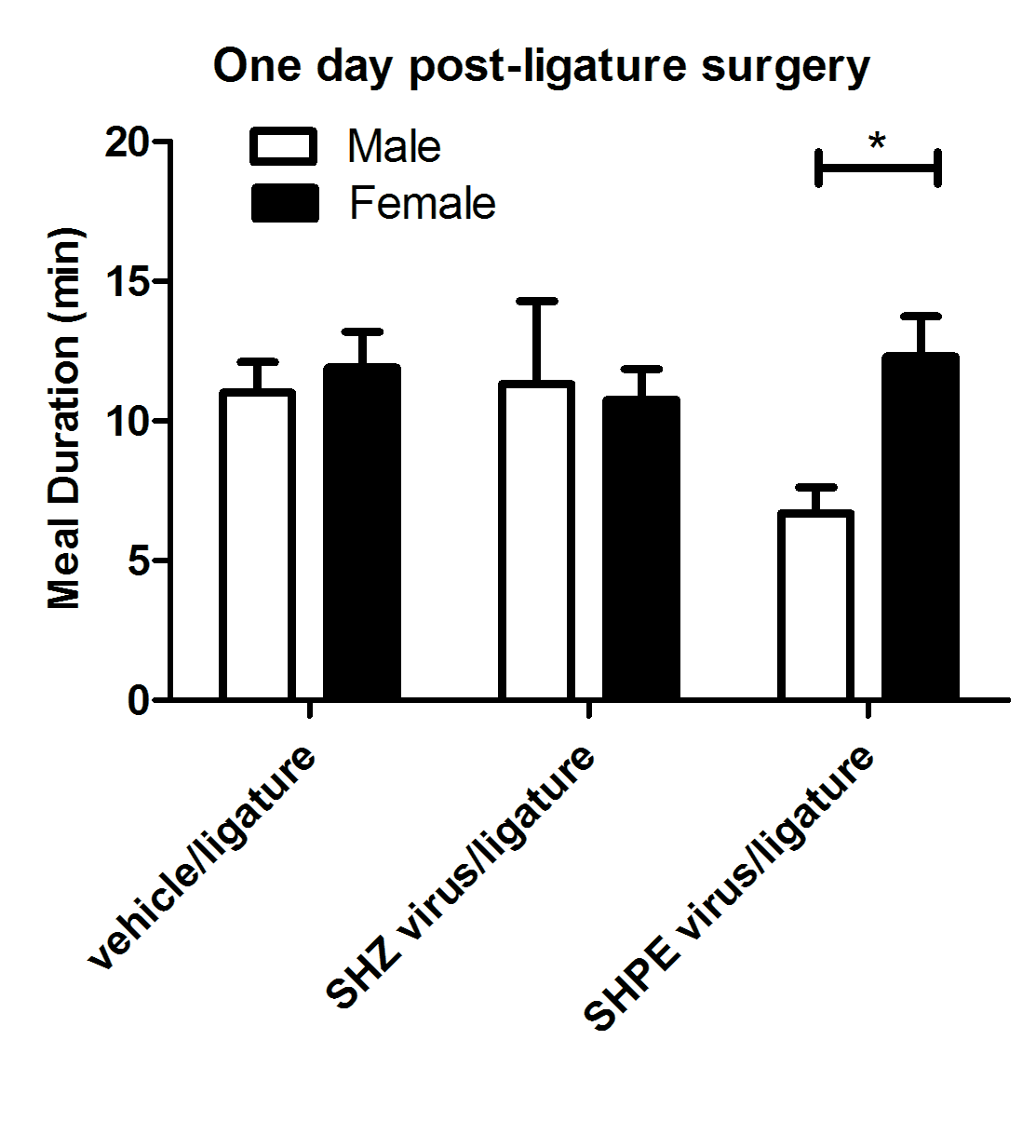


**Additional file1: Figure S1. Experiment #2: Meal duration for male and female rats one day after ligature surgery.** Rats were injected with virus and then 72 hours post-injection a ligature was placed around the tendon of the masseter muscle. Meal duration is reported for the day after surgery. Groups received either a vehicle injection or an injection of virus that did not contain an enkephalin transgene (control virus, SHZ) or a virus that had an enkephalin transgene (SHPE) 72 hours before ligature surgery. A significant difference (P<0.05, t-test) between the male and females was observed in rats that received the SHPE virus (asterisk). See table two for the number of animals per group.
